# Integrated review of the knowledge, attitudes, and practices of maternity health care professionals concerning umbilical cord clamping

**DOI:** 10.1111/birt.12647

**Published:** 2022-05-18

**Authors:** Lisa Peberdy, Jeanine Young, Debbie Massey, Lauren Kearney

**Affiliations:** ^1^ University of the Sunshine Coast Sunshine Coast Queensland Australia; ^2^ Southern Cross University Lismore New South Wales Australia; ^3^ University of Queensland Brisbane Queensland Australia

**Keywords:** cord clamping, integrative review, midwives, obstetricians, placental transfusion

## Abstract

**Background:**

Umbilical cord clamp timing has implications for newborn health, which include increased iron stores up to 6 months of age. National and International cord clamping guidelines differ as do health professionals' practices. The rationale for differences in cord clamping practice is unclear.

**Aims and objective:**

Studies on the knowledge, attitudes, and practices of maternity health care professionals about cord clamp timing were synthesized. Similarities and differences between professional groups and understanding of the optimal timing of cord clamp timing for term newborns were compared.

**Methods:**

An integrative review was undertaken. PubMed, Scopus, MIDIRS, CINAHL, and Google Scholar were searched. Publication date limits were set between January 2007 and December 2020. Quality appraisal was undertaken using the Critical Appraisal Skills Program (CASP) tools.

**Results:**

Eighteen studies met inclusion criteria, as they included primary research studies that investigated maternity health care professionals' knowledge, attitudes, and practices about umbilical cord clamping, and were written in English. Four main subject areas were identified: a) knowledge of optimal cord clamp timing; b) attitudes and perceptions of early vs deferred cord clamping; c) cord clamping practice; and d) rationale for cord clamping practice.

**Conclusions:**

Different attitudes and practices were identified between midwifery and medical professionals in relation to cord clamp timing together with health professional knowledge and practice gaps pertaining to optimal cord clamp timing. Contemporary evidence should inform guidelines for clinical practice and be embedded into maternity health professional curricula and professional development programs.

## 1 INTRODUCTION

The World Health Organization^1^ recommends active management of the third stage of labor (AMTSL) includes administration of a uterotonic (if available) after birth of the infant, deferred cord clamping, and placental delivery by controlled cord traction, followed by uterine massage. Physiological or expectant third‐stage management is defined by the UK National Institute of Clinical Excellence (NICE) as no administration of a uterotonic drug, no clamping of the cord until pulsations cease, and placental delivery by maternal effort and gravity.^2^

Cord clamp timing (CCT) in active management of the third stage of labor (AMTSL) varies in the UK and internationally. Initially, early cord clamping (ECC) was introduced as part of AMTSL without evidence of postpartum hemorrhage (PPH) prevention.^3^ Deferred cord clamping (DCC)—also referred to as “delayed” cord clamping—is now recommended as part of active management, and as such, its inclusion in AMTSL may be referred to as modified active management of third stage.^1^

No consistent definition of early, deferred, or optimal cord clamping exists.^4^ International, national, and local guidelines, protocols, and individual clinician practice differ.^5^ ECC is generalized as clamping before one minute postbirth, and DCC is generalized as clamping from one minute postbirth through until the cord stops pulsating in two large CCT systematic reviews.^6^, ^7^ An intermediate category of CCT has been identified as clamping between 30 seconds and 2 minutes postbirth.^8^ Deferred cord clamping is now accepted and supported by international professional bodies; however, the term “delayed or deferred” cord clamping in one recommendation is not always consistent with another recommendation (see Table 1). These discrepancies may cause confusion in clinical practice.

**TABLE 1:** Summary of current International and National Guidelines and Recommendations for optimal cord clamping

Year	Guideline	Recommendation
2017	American College of Obstetricians and Gynecologists (ACOG): Committee Opinion: Delayed Umbilical Cord Clamping after Birth.	“Given the benefits to most newborns and concordant with other professional organizations, ACOG now recommends a **delay** in umbilical cord clamping in vigorous term and preterm infants for at least 30‐60 s after birth” (Page 1).
2017	National Institute of Clinical Excellence (NICE): Intrapartum Care for Health Women and Babies.	“Do not clamp the cord earlier than 1 min from birth of the baby unless there is concern about the integrity of the cord or the baby has a heart rate less than 60 beats/min that is not getting faster.” “Clamp the cord before 5 min in order to perform controlled cord traction as part of active management.” “If the woman requests that the cord is clamped and cut later than 5 min, support her in her choice.”
2016	Australian and New Zealand Council of Resuscitation (ANZCOR). Guideline 13.1: Introduction to Resuscitation of the Newborn.	We suggest **delayed** cord clamping for preterm infants not requiring immediate resuscitation after birth. (Nil time range provided with definition of delayed cord clamping) (Page 7).
2015	Royal College of Obstetricians and Gynecologists (RCOG): Clamping the Umbilical Cord and Placental Transfusion. Scientific Impact Paper No.14.	In healthy term babies, evidence supports **deferring** clamping of the umbilical cord, as this appears to increase iron stores in infancy. This assessment of the evidence in concordant with the Cochrane review and recommendations by NICE (Page 7). Suggest the term ***Immediate*** cord clamping to be used to mean within 30 s of birth. ***Deferred*** cord clamping defined as not until at least 2 min post birth. ***Intermediate*** cord clamping could refer to clamping the cord between 30 s and 2 min (Page 2). The Royal College of Obstetricians and Gynecologists (2015) prefer the term deferred as it suggests a planned policy as opposed to delayed which might imply later than ideal (Page 2).
2015	International Liaison Committee on Resuscitation (ILCOR). Neonatal Resuscitation Part 7: Specific treatment recommendations.	“For uncompromised babies, a **delay** in cord clamping for at least 1 min after complete delivery of the infant is now recommended for term and preterm babies. As yet, there is insufficient evidence to recommend timing of cord clamping in compromised newborns who require resuscitation” (Page 252).
2014	Royal Australian and New Zealand College of Obstetrics and Gynaecology (RANZCOG): Provision of routine intrapartum care in the absence of pregnancy complications.	Term infants: **Delayed** cord clamping is associated with increased hematocrit and decreased iron deficiency at 3‐6 months. These benefits are achieved at the expense of an increased risk of early polycythemia and jaundice. 75% of placental transfusion volume occurs in the first minute. At present, no clear evidence to guide practitioners regarding **delayed** cord clamping in term infants, but infants most likely to benefit are those where maternal iron stores are low, or in infants who will be exclusively breastfed without iron supplements (Page 11).
2014	World Health Organization (WHO). Guideline: Delayed umbilical cord clamping for improved maternal and infant health and nutrition outcomes.	**“Delayed** cord clamping (not earlier than 1 min post birth) is recommended for improved maternal and infant health and nutrition outcomes” (Page 10).
2014	American College of Nurse Midwives Position Statement: Delayed Umbilical Cord Clamping.	**Delayed** cord clamping should be standard of care in all birth settings for term newborns (Page 1).
2012	Royal College of Midwives (RCOM). Evidence Based Guidelines for Midwifery‐led care in Labor: Third Stage of Labor.	**Delayed** cord clamping is currently the recommended practice known to benefit the neonate in improving iron status up to 6 months, but with a possible risk of jaundice that requires phototherapy (Page 2).
2012	QLD Maternal and Newborn Clinical Guidelines (Australia). Normal Birth.	“Physiological Management: Clamp cord after pulsations cease (Page 6 & 34); Active management: **Early** clamping and cutting of cord (Page 35); Modified Active Management: Clamp cord once the cord pulsation ceases (Page 6), clamp the cord close to the perineum. Earlier clamping when the newborn requires extensive resuscitation measures” (Page 35).
2011	International Federation of Gynecology and Obstetrics (FIGO): Delayed cord clamping to prevent newborn problems.	**Immediate** cord clamping is no longer recommended. “Maternal and newborn health researchers have recommended delaying the clamping of the umbilical cord after birth” (Page 1).
2010	Resuscitation Council UK: Newborn Life Support Guidelines (NLSG).	“For uncompromised babies, a **delay** in cord clamping of at least 1 min for the complete delivery of the infant is now recommended” (Page 119).
2009	Society of Obstetricians and Gynecologists Canada (SOGC): Active Management of the Third Stage of Labour: Prevention and Treatment of Postpartum Haemorrhage.	“When possible, **deferred** cord clamping by 1 min for preterm infants under 37 weeks. For term infants, the possible increased risk of jaundice requiring phototherapy must be weighed against the physiological benefit of greater hemoglobin and iron levels up to 6 months of age as a result of deferred cord clamping” (Page 1).

Deferred cord clamping provides higher initial hemoglobin concentrations, and increased iron stores up to 6 months of age for infants.^6^, ^7^, ^9^ Furthermore, it has been associated with improved scores in fine‐motor and social domains at 4 years of age in infants born after a low‐risk pregnancy, especially in boys,^10^ affirming the critical nature of this clinical practice: that clamp timing affects neurodevelopment in a low‐risk population of children born in a high‐income country.^10^

The effects of CCT for term infants on maternal and neonatal outcomes were explored in a 2013 systematic review of 15 randomized trials.^6^ McDonald and colleagues reported fewer infants in the ECC group required phototherapy for jaundice than those in the DCC group.^6^ Maternal postpartum hemorrhage risk was not affected by the timing of cord clamping^6^ despite being the original rationale for inclusion of ECC as part of AMTSL.^11^

Cord clamp timing may be influenced by parental desire for collection of cord blood for donation or private storage options. Cord blood stem cells can be used as an alternative to bone marrow stem cells to treat hematological, immunological, and genetic disorders.^12^ The need for cord blood gas sampling soon after birth may also affect health care professionals' decision making about CCT. A 2013 randomized trial to investigate the effect of DCC compared with ECC on maternal postpartum hemorrhage (PPH) and umbilical cord blood gas sampling of 382 term births after a low‐risk pregnancy found no significant effect on the proportion of viable cord blood gas samples as a result of DCC.^13^

Health care professionals' current CCT practice may be based on individual preference^14^ and outdated knowledge.^15^, ^16^ Changing recommendations and poor understanding of the optimal timing of cord clamping make it difficult for health professionals to provide evidence‐based care and inform women of best practice options for their infant's care during the third stage of labor. Cord clamping is often left to individual practitioners' preference and hospital routines,^14^ despite the importance of this practice on neonatal outcomes.

As such, the purpose of this project was to examine health care professionals' knowledge, attitudes, and practices concerning CCT in order to identify gaps in knowledge that may lead to confusion, ambiguity, and inconsistency in practice.

## 2 AIMS AND METHOD

This review, first, aimed to identify studies of maternity health professionals' knowledge, attitudes, and practices of cord clamp timing in response to changing recommendations, and factors influencing the practice of cord clamp timing. Second, we compared findings between professional groups relating to optimal cord clamp timing.

### 2.1 Search methods

The integrative review (IR) method was chosen for this review.^17^ IRs permit and facilitate a holistic evaluation of the strength and limitations of published evidence using a combination of diverse methodologies.^18^ The five‐stage model,^17^ comprising problem identification, literature search, data evaluation, data analysis, and presentation,^18^ provided a framework to guide this integrative review and enabled rigorous evaluation of the strength of the evidence and identification of gaps in the literature exposing the need for further research.^18^ The PRISMA checklist was chosen as the reporting guideline. See File S1.

Databases searched included PubMed, Scopus, MIDIRS, CINAHL, and Google Scholar using search terms: cord clamping, third stage labor management, maternity care practitioners, obstetricians, midwives, knowledge, attitudes, and practices. Publication date limits were set between January 1, 2007, and December 31, 2020, to identify studies conducted during a period of international change in recommendations for CCT.

### 2.2 Literature search

Inclusion criteria were primary research studies that investigated and reported maternity health care professionals' knowledge, attitudes, and practices about umbilical cord clamping, and written in English using quantitative, qualitative, or mixed‐methods designs. Cord clamping practices relating to vaginal birth of term newborns were the focus of the literature search, given that the majority of births are contained in this group; however, consideration was given to clamping practices relating to babies born preterm and/or by means of cesarean where these were reported specifically in the literature. Studies that reported health professional knowledge, attitudes, and practices about umbilical cord clamping, which did not differentiate birth mode or gestational age‐specific practices, were included in this review when these publications were directly relevant to the primary research question posed. The initial search was conducted by LP who identified potential papers for inclusion based on their titles and abstracts, with all papers for inclusion agreed on through consensus by JY, DM, and LK.

**FIGURE 1:**
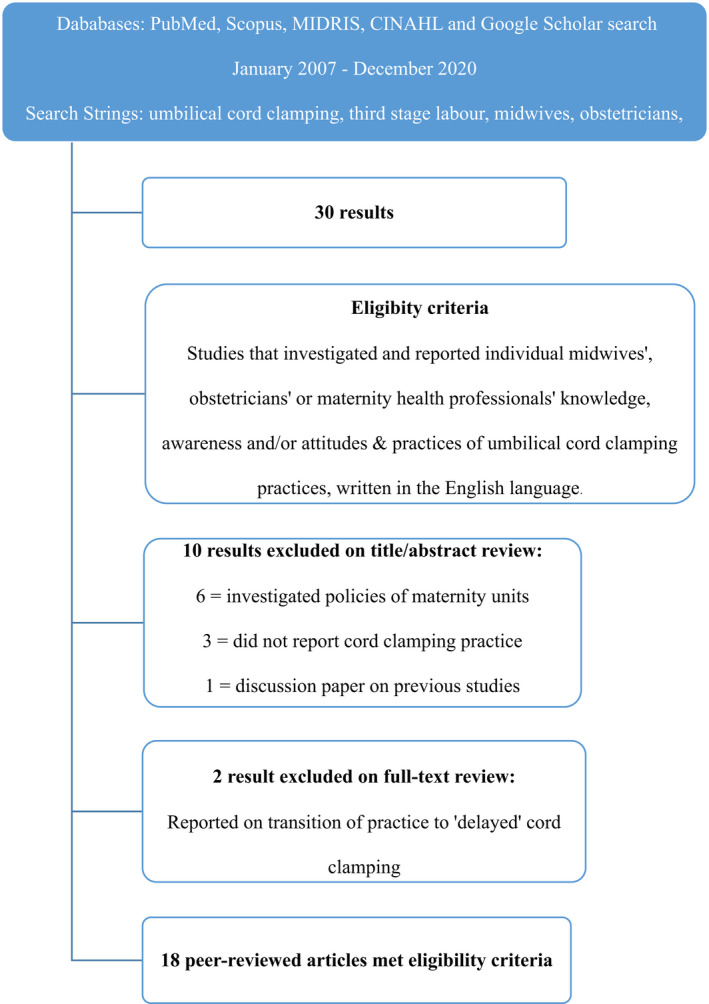
Screening and inclusion process [Color figure can be viewed at wileyonlinelibrary.com]

Exclusion criteria included papers not available in English, discussion papers, and papers reporting on individual maternity unit standard practice or policies on cord clamping. Figure 1 details the structured search conducted, including the search strategy and inclusion process applied to the peer‐reviewed literature.

### 2.3 Data evaluation (data extraction and synthesis)

Each article was read and summarized to identify key points and common themes relating to maternity health professionals' knowledge, attitudes, and practices about CCT. After identification of themes, the studies were subgrouped by maternity health professional group. Findings and variables were grouped together, and similarities and differences between studies were compared.

### 2.4 Data analysis (quality appraisal)

Critical Appraisal Skills Program (CASP) tools were used to determine study quality.^19^ Quantitative and mixed‐methods studies were assessed using the CASP Cohort Study Checklist. Qualitative studies were assessed using the CASP Qualitative Checklist. The quality appraisal examined each included study, evaluating research aims, methods, analysis, results, and discussion. No studies were excluded after assessment of validity and quality. CASP assessments are shown as a File S1.

A total of 30 articles were identified that provided description relating to maternity health professionals' knowledge, attitudes, or practice of umbilical cord clamping. Twelve papers were excluded because they did not meet inclusion criteria or the aims of this integrative review.^20^, ^21^, ^22^, ^23^, ^24^, ^25^, ^26^, ^27^, ^28^, ^29^, ^30^, ^31^ One short report of original research findings that provided minimal yet sufficient methodological detail^32^ was also included because the study aims and results were directly relevant to this review. Analysis of eligible studies identified four main subject areas through a constant comparison method; an overarching approach in the development of the results in IRs.^17^ This involved analysis of studies where the data were extracted into systematic categories, identifying distinct patterns, themes, and relationships within and across the studies. Because of the range of study designs included, and heterogeneity of primary outcome measures reported, quantitative synthesis could not be conducted as part of this integrative review.

### 2.5 Samples and settings

This search of international literature identified 18 eligible studies. Reference lists of selected articles were also examined for relevant publications, which met review inclusion criteria.^11^, ^14^, ^15^, ^16^, ^32^, ^33^, ^34^, ^35^, ^36^, ^37^, ^38^, ^39^, ^40^, ^41^, ^42^, ^43^, ^44^, ^45^ Empirical studies selected for this review used quantitative (n = 15) and qualitative designs (n = 3). Cross‐sectional survey design was frequently used (n = 13)^14^, ^15^, ^16^, ^32^, ^33^, ^34^, ^35^, ^36^, ^39^, ^40^, ^43^, ^44^, ^45^; and/or observational audit (n = 4)^33^, ^34^, ^37^, ^38^ to elicit and describe knowledge, attitudes, and or practices relating to umbilical cord clamping. Cross‐sectional survey and audit studies varied in sample size (27‐1243 participants) with most studies recruiting from several maternity professional disciplines (see Table 2). Focus groups using multidisciplinary (n = 22)^11^ and midwife‐only (n = 10)^42^ samples were used to explore third‐stage labor management including cord clamping practices in two studies. Two qualitative studies reported on multidisciplinary cord clamping perceptions and practice.^41^, ^42^

**TABLE 2:** Study characteristics and key findings (cord clamping findings only) for included review papers (n = 18)

Author/Year	Aim	Country/setting	Sample/inclusion	Design	Findings	Strengths/limitations
Quantitative‐descriptive observational studies (cross‐sectional survey)						
Peberdy et al (2020).	To identify health professional knowledge and attitudes toward third‐stage labor options of cord clamp timing, cord blood banking, and donation and their practice of informing parents of these options	Australia National Professional organizations Public and private settings	Total n = 129 Midwives: 105 Obstetricians: 24	Cross‐sectional self‐administered survey (electronic) Questionnaire n = 51 items	**Health professional differences in knowledge, attitudes, and practices relating to CTT** *Knowledge*: Identify ECC as within 1 min (Obs 95.5% Vs MW 71.7%, *P* = .048); Choose correct DCC definition (MW 68% Vs Obs 50%, *P* = .002). *Attitudes*: Importance of providing parents with information about: Placental transfusion: MW 87.4% vs Obs 37.5%, *P* < .001; CCT on infant health: MW 90% vs Obs 70.8%, *P* < .001; CCT on maternal health: MW 98% vs Obs 70.8%, *P* < .001; Options for CTT: MW 94.1% vs Obs 50%, *P* < .001. *Practices*: Discuss CCT with all clients: MW 79.6% vs Obs 20.8%, *P* < .001, support parent preferences for CCT: MW 99% vs Obs 78.3%, *P* < .001; clamp cord after pulsations cease: MW 82.7% vs Obs 72.7%, *P* < .001.	*Limitation*: Convenience sampling, lower representation from obstetricians than midwives; most participants from one Australian state may limit generalizability. *Strengths*: First study in Australia to assess CCT attitudes and practice. Used a validated instrument to collect data.
Ibrahim et al (2017). Current umbilical cord clamping practices and attitudes of obstetricians and midwives toward delayed cord clamping in Saudi Arabia.	To investigate cord clamp timing practice, perceptions, and attitudes. *Term* and *preterm*	Saudi Arabia Riyadh 5 tertiary hospitals	Total n = 157 Midwives: 75 Obstetricians:82 RR = 80%	Cross‐sectional self‐administered survey Questionnaire n = 24 items	*Positive attitudes for DCC*: MW 34.4% (n = 21/61) Obs 38% (n = 27/71) *CC practice—no set time*: MW 30.6% (n = 22/72) Obs 48.7% (n = 38/78) *Combined results for term CCT practice*: Followed protocol, 35.7% (n = 56) No set reason 26.1% (n = 41) After pulsations cease, 20.4% (n = 52) *ECC reasons*: Always clamp early, 33.1% (n = 52) Parents wish, 3.8% (n = 6) *DCC reasons*: Cord still pulsating, 26.1% (n = 41) Parents wish, 12.1% (n = 19) *Combined results for preterm CCT practice*: No set time, 65.6% (n = 103) No set reason, 19.1% (n = 30) Same reason as for term infants, 21.7% (n = 34) DCC benefits 3.8% (n = 6)	*Limitation*: Convenience sampling may limit generalizability. *Strengths*: First study in region to assess CCT attitudes and practice. Used a validated widely used instrument to collect data.
Schorn et al (2017). USA Physician and Midwife Adherence to Active management of the Third Stage of Labor International recommendations	To determine routine patterns for managing the third stage of labor in the USA. To provide a national description of practices used during the third stage.	USA National Study Varied settings	Total n = 1243 Obstetricians and physicians: 368 Midwives: 875	Quantitative Cross‐sectional survey Postal questionnaire n = 23 items	Participants self‐selected best description category of their usual cord clamp practice: *Clamped within 1 min*: 18.3% (221) 6.4% (54) MWs 46% (167) Obs *Clamped after 1 min*: 81.7% (987) 93.6% (791) MWs 54% (196) Obs	*Limitations*: Low response rate: 13% Self‐reported patterns of practice: not as reliable as observational studies that directly monitor clinicians' behaviors and may distort some estimates. *Strength*: Good geographical distribution.
Jelin et al (2014). Obstetricians' attitudes and beliefs regarding umbilical cord clamping.	To assess obstetricians' attitudes and beliefs about cord clamping	USA	n = 176 practicing obstetricians. Obstetric members of American College of Obstetricians and Gynaecologists (ACOG), n = 83; RR = 20.8% and Collaborative Ambulatory Research Network (CARN) n = 93 RR = 46.5%	Quantitative Cross‐sectional Survey Questionnaire: No. of items not stated	3.5% had a DCC policy at the hospital where they practiced. *Is UCC timing important*: Respondents reported UCC timing very important for preterm under 28/40 though less important with increasing gestation. *What is the optimal timing of UCC*: 42%‐55% were unaware, 19.9% said immediately after milking for preterm under 28/40, 20.9% said after 1 min for those over 36 weeks. *Do situations affect recommended UCC time*: 69.9% said if immediate resuscitation was required, 52.6% said if placental abruption was required, and 30.6% said if placenta praevia was required. *Summary*: Majority of respondents would ECC for maternal hemorrhage or neonatal resuscitation, and DCC in preterms for blood transfusion purposes. Obs who believe DCC to be important had opinions about the risks and benefits inconsistent with current literature.	*Limitation*: Survey not validated
Stoll & Hutton (2012). A survey of umbilical cord clamping practices and attitudes of Canadian maternity care providers.	To understand how maternity care practitioners' interpret evidence around cord clamp timing and what they do in practice.	Canada National study	n = 353 maternity care practitioners: MW (190, 54%); Obs (85, 24%); GPs (74, 21%) Maternal/fetal specialists (4 1%). Members of Canadian Association of Midwives (CAM) and Society of Obstetricians and Gynaecologists (SOGC), and Family Physicians Eligibility criteria: provision of intrapartum care at time of survey	Quantitative Cross‐sectional survey Online questionnaire: No. of items not stated	*Clamped cord within 30 s*: *(Term)* Obs – 77.9% Phys – 60% MWs – 9.8% *Clamped cord at 2 min or more*: *(Term)* Obs – 6.5% Phys – 9.1% MWs – 65.7% MWs more likely to make a conscious decision re UCC time. Most common reason for ECC (term): 70% resuscitation or medical intervention. Results highlight interprofessional variations in cord clamp practices.	*Limitations*: Low response rate, especially for physicians Midwives over‐represented. Actual cord clamping practices may differ from self‐reported practice.
Sivaraman & Arulkumaran (2011). Delayed cord clamping: potential for change in obstetric practice	To investigate obstetricians and midwives' perceptions of early vs delayed umbilical cord clamping across 4 London Trust Hospitals	UK 4 large London hospitals	n = 148 maternity staff of 4 South London Hospitals: [19 Obs consultants; 20 Obs registrars; 9 specialist fellows; 76 MWs, 24 student midwives].	Quantitative Cross‐sectional survey Questionnaire: No. of items not stated	53% clamped between 20 and 40 s, 80% clamped before 40 s Most knew some disadvantages of ECC such as anemia/possible anemia‐related neurological development delay but only 2% DCC after 2 min. 64% did not believe there was a relationship between ECC and less frequent PPH.	*Limitation*: Editorial summary of study only.
Farrar et al (2010). Care during the third stage of labour: a postal survey of UK midwives and obstetricians.	To understand more about current third stage labor care in the UK.	UK National study	n = 1194 Obstetric members of RCOG: 53% Response Rate (RR), n = 1194/2230) 78% (n = 926) conducted or supervised births in the last 12/12. n = 1702 Midwifery members of the RCOM, UK. 71% RR (n = 1702/2400). 76% (n = 1297) conducted or supervised a birth in the last 12/12.	Quantitative Cross‐sectional survey Postal questionnaire n = 16 items Postal questionnaire approved by RCOG & RCOM. Pilot study of 12. Survey tested, modified, and retested.	*AMTSL*: 93% of Obs & 73% MWs always/usually practiced AMTSL for vaginal births. *UCC*: 41% MWs and 74% of OBs UCC at 20 s for term infants. *LSCS*: UCC rare after 60 s. *Overall*: UCC: varies between MWs and Obs. Obs mostly UCC within 1 min for both vaginal and LSCS births. No consensus on ECC v DCC definitions. Same ECC v DCC definitions given by both groups. High level of no responses to questions on timing may reflect uncertainty in definitions. Both Obs and MWs thought more evidence needed from trials to guide 3rd‐stage care.	*Limitation*: Low response rate of Obs. Drs current practice for 3rd stage is unlikely to have influenced willingness to respond. *Strength*: High response rate from MWs, representative of the UK practice.
Downey and Bewley (2010). Childbirth practitioners' attitudes to third stage management.	To examine third‐stage management beliefs and practices of midwives, obstetricians, and neonatologists in one London Foundation Trust.	UK	n = 73 active obstetric, midwifery, and neonatal staff at a large London NHS Hospital. [45 midwives 19 obstetricians 9 neonatologists]	Quantitative Cross‐sectional survey Electronic and mail Self‐reported questionnaire n = 14 MC items Piloted and amended.	21% of obs & 38% of MWs define ECC within 1 min. Anemia was most commonly thought to be associated with ECC. 77% of respondents defined DCC as when the cord stopped pulsating. Jaundice and polycythemia most commonly cited as associated with DCC. 76% stated resuscitation as an indication of ECC. 44% cited UCC after 60 s was to allow placental transfusion. 41% of respondents consider woman's preference for 3rd‐stage management, which reflected hospital recommendation *UCC in AMTSL*: 63% of Obs and 57% of MWs UCC within 60 s despite having a local DCC guideline recommendation. *UCC in Expectant management*: 69% MWs and 74% Obs UCC after pulsations ceased. Gaps exist in the knowledge about possible consequences of ECC and DCC. 60% of respondents said current UCC guidance was inadequate; therefore, more guidance as to optimal UCC time was required.	*Limitations*: Low response rate: 26% (73/284). Results from one large London hospital only so may not be generalized. Results in graphs are difficult to analysis.
Ononeze & Hutchon (2009). Attitude of obstetricians towards delayed cord clamping: a questionnaire‐based study.	To ascertain whether obstetricians adopted recommendation to delay cord clamping by 2 min in preterm infants.	International	Obstetricians (n = 43) attending 11th Annual conference of the British Maternal & Fetal Medicine Society 2006. Representatives from the UK, EU, the USA, Canada, Australia	Quantitative Cross‐sectional survey Questionnaire n = 2 items	9.3% always performed DCC. 53.4% performed DCC on some occasions. 37.2% never performed DCC. *Reasons for noncompliance*: 78.2% difficult to do in practice; 8.6% unaware of evidence; 13% no reason given. *Those who never complied*: 50% unaware of evidence; 37.5% difficult to do in practice; 6.2% do not believe the evidence; 6.2% no reason indicated. *Discussion*: DCC is feasible. Resuscitation can begin at bed and allows extra placental transfusion. Opposing view: DCC alters cord pH, which may have medico‐legal implications for practitioner. *Results*: Obs reluctant to practice DCC as unaware of scientific knowledge despite proven benefits of this practice.	*Limitation*: Very little methodological detail and information recorded. *Strength*: 100% response rate so a good indication of current thoughts and practices.
Tan et al (2008). How do Physicians and midwives manage the third stage of labor.	To learn about physician and midwives third stage management, identify factors that may influence their management choice, and to understand reasons for not following active management of third stage.	Canada British Columbia	n = 77 obstetricians (British Columbia College of Physicians & Surgeons members) n = 163 family physicians. n = 47 Midwives (College of Midwives of British Columbia members) Response rate = 45.8%	Quantitative Cross‐sectional survey Postal questionnaire n = 14 items Multiple choice and short answer	MWs less likely to perform ECC **Usual cord clamp timing practice:** *Early*: Obs (n = 60; 77.9%); physicians (n = 114; 69.9%); MWs (n = 1; 2.2%) *Late*: Obs (n = 1; 1.3%); Physicians (n = 6; 3.7%); MWs (n = 28; 60.9%) *Variable*: Obs (n = 16; 20.8%); Physicians (n = 41; 25.2%); MWs (n = 16; 34.8%)97.8% midwives, 85.3% of Obstetricians, & 53.7% of family physicians were familiar with the current guidelines. 3 MW commented on the inappropriateness of early cord clamping as a reason why they rejected full active management guidelines.	*Strength*: Questionnaire pretested for comprehension and validity. *Limitations*: Only studied British Columbia maternity caregivers; therefore, results may not be generalized across Canada. Study relied on self‐reporting of practice, attitudes and behavior, no verification if respondents practiced in reported manner.
Mixed methods (cross‐sectional survey and observational audit)						
Ortiz‐Esquinas et al (2020). Variability and associated factors in the management of cord clamping and the milking practice among Spanish obstetric professionals.	To determine the variability in cord clamping practice. To identify factors of cord clamping management.	Spain National study	Total n = 1045 Midwives (789, 75.5%) Obstetricians (115, 11%) Student midwives or obstetricians (141, 13.5%)	Cross‐sectional survey Online questionnaire n = 35 items Observational study	*Most professionals perform DCC*: 92.2% (n = 964) Waited 1‐2 min: 14.1% (n = 147) Clamp after pulsations ceased: 69.3% (n = 724) MW: 96.5% (n = 855) Obs: 68.6 (n = 109) *Overall ECC*: (n = 81) MWs: 3.5% (n = 31) Obs: 31.4% (50).	*Limitations*: Selection bias – greater number of midwifery as opposed to obstetric participants. *Strengths*: First study conducted in Spain. Large study that reveals variability among professionals.
Airey et al (2008). Timing of Umbilical Cord Clamping: midwives' views and practice	To observe current practice of third stage at Bradford Royal Infirmary.	UK Birth Suite of a large inner‐city hospital	n = 52 midwives surveyed n = 100 births observed for cord clamp timing	Quantitative Cross‐sectional survey Questionnaire n = 6 items Observational audit n = 100 births	92% said their usual practice was to clamp within 1 min. 75% defined ECC as immediately after birth. 80% defined DCC as when pulsations ceased. *Practice*—85% clamped within 30 s, 32% clamped within 10 s. 4 infants had their cord clamped after more than 1 min. *Discussion*—Factors in UCC appeared to include MW experience, presence of a 2nd MW, and speed of birth. Impression is MWs do not plan DCC, rather ECC not always possible. Main benefit of DCC is infant well‐being, though it may also drain placental circulation and facilitate placental separation, shorten 3rd stage and reduce risk of PP blood loss. *Conclusion*: MWs currently practice ECC with variation in when UCC occurs.	*Limitation*: Study conducted in one facility therefore cannot be generalized.
Bimbashi et al (2010). Care during third stage of labour: obstetrician views and practice in an Albanian maternity hospital.	To understand obstetricians' views and observe current third stage labor practice.	Albania 1 large tertiary referral hospital in Tirana.	n = 27 obstetricians	Quantitative *Mixed methods*: Cross‐sectional Survey and Observational Audit Questionnaire: n = 6 items Audit Tool: N = 8 items	Variations found with UCC although most clamped within 1 min. With physiologic care, 42% (8/19) clamped within 20 s. Practice observed for 156 births: (27% = LSCS, 10% before 37/40, timing not recorded in 20% of births). UCC usually within 20 s; UCC occurs within 30 s in 90% of births; in all births by 50 s. *LSCS UCC*: 11‐20 s (86%, 36/42) 21‐30 s (7%, 3/42) 31‐40 s (5%, 2/42) 41‐50 s (2%, 1/42) (6%, 1/16). Some Obs reported they waited longer than 60 s to clamp but this was not observed. 93% (27/29) thought there should be trials comparing ECC v DCC.	*Strength*: Questionnaire and audit tool adapted from previous study (Airey et al, 2008).
Observational audits						
Hutton et al (2012). An observational study of umbilical cord clamping practices of maternity care providers in a tertiary care centre.	To investigate actual cord clamping time and circumstances at a large Tertiary Canadian Hospital	Canada 1 Large Tertiary British Columbia Womens' Hospital	n = 101 practitioners (midwives, family physicians, and obstetricians) Births observed by profession group (n = 89): [Obstetricians: 39; GPs: 37; Midwives: 13	Quantitative Descriptive observational audit of cord clamp timing Data collection by a research nurse. 100 births of 96 singleton infants and 2 sets of twins.	*Findings*: 56.2% (50/89) of UCC at or before 15 s, the median UCC time was 12 s. *Time to clamp*: 0‐15 s: Obs 66.7% (n = 26); GPs 48.6% (n = 18); MWs 46.2% (n = 6) 16‐60 s: Obs 25.6% (n = 10); GPs 18.9%(n = 7); MWs 0 Over 2 min: Obs 0; GPs 13.5% (n = 19); MWs 38.5% (n = 5) Obs more likely to clamp immediately. 45% of all HCPs clamped before 15 s. *Median UCC time*: Obs = 12/60, GPs = 19/60, MWs = 81/60. Median UCC time more likely to be longer when spontaneous birth, in low‐risk unit, no UCB collection, and no birth or infant complications.	*Limitations*: Single institution and small sample size so may not be representative to all HCPs. Practitioners may have altered typical practice of UCC because of being observed.
Blouin et al (2011). Effects of a two‐component intervention to change hospital practice from early to delayed umbilical to delayed umbilical cord clamping in Peruvian Amazon.	To investigate the effect of a two‐component intervention to change hospital practice about the timing of cord clamping.	Peru	n = 224 births with nurses and midwives in attendance (Pre n = 112, Post n = 112)	Quantitative **Pretest post‐test intervention Design** Intervention consisted of 1) 3‐day best practice training workshop on birthing, and 2) a hospital directive. Data Collection: Audit of cord clamp timing pre and posteducation intervention (measured time from birth of 1st shoulder to clamping of cord)	n = 112 cord clamping events. Preintervention mean UCC time: 56.8 s (8.9‐191.7 s). Postintervention mean UCC time (n = 112): 169.8 s (13.4‐397.3 s). UCC greater than 1 min increased from 39.3% (pre) to 85.7% (post). UCC less than 2 min decreased from 95.5% (pre) to 95.5% (post). Education was associated with improved practice consistent with evidence. *Summary*: To change practice successfully, health professionals need to be made aware of the scientific evidence.	*Limitations*: Presence of researchers observing practice may have altered practice of nurse‐midwives. Postimplementation data collection from intervention collected at a later date may have been more informative of long‐term practice change/modification.
Qualitative studies: Focus groups and Interviews						
Peberdy et al (2020). Maternity health professionals' perspectives of cord clamp timing, cord blood banking and cord blood donation: a qualitative study.	To explore Maternity health professionals' perspectives toward CCT (and CBB, CBD)	Australia National Professional organizations Public and Private settings	n = 14 maternity health professionals. MW n = 11 Obs n = 3	Qualitative (Phase 2 of a mixed‐methods study) Semi‐structured interviews Purposive sampling Thematic analysis	3 Themes identified in relation to HCP perceptions and practice of CC. MWs placed greater value on informing /discussing CCT with parents then Obs. Despite broad support for DCC, a lack of consistency as to what constituted DCC. DCC was not guided by specific time frame, was dependent on individual perception of DCC. Clinical scenario was a factor in influencing CC practice.	*Limitations*: Limited representation of obstetricians (21%). Majority of participants (71%) from 1 state only. *Strengths*: First Australian study on third‐stage labor perspectives. Use of semistructured interview to collect and cover all required material.
Mwakawanga & Mselle (2020). Early or delayed umbilical cord clamping? Experiences and perceptions of nurse‐midwives and obstetricians at a regional referral hospital in Tanzania.	To describe CCT experiences and perceptions at a regional referral hospital in Tanzania.	Tanzania Secondary level regional referral hospital	Total n = 19 Interviews: MW n = 6 Obs n = 3 Discussion group: MW n = 10	Qualitative Semi‐structured interviews and focus group discussions. Purposive sampling, thematic analysis	3 Themes identified *Experiences of CCT*: CC <1 min occurs immediately or after infants' 1st breath CC after pulsations cease *Perceptions of CCT*: Benefits outweigh risks *Factors influencing CCT*: Knowledge, presence of guidelines, adequate human resources, and equipment.	*Limitations*: Interviewer with specialty knowledge may have introduced bias and influenced interpretation of results. *Strengths*: Triangulation increased validity and reliability. Data analysis attended by both authors.
Schorn et al (2015). An exploration of how midwives and physicians manage the third stage of labor in the USA.	To identify practitioner reported assessments and interventions used during the third stage of labor, and to examine which management steps practitioners believe should always be used in third stage of labor.	USA	n = 22 Birth attendants (nurse‐midwives, professional midwives, obstetricians, and GPs) who attended at least 1 birth in the last year.	Qualitative Focus groups (40‐ to 60‐min duration) using a modified group technique to avoid unconscious group‐censoring effects. Purposive sample	MWs noted patient preferences that would affect management: mother's desire for DCC, lotus birth, placental ingestion. Intrapartum tasks that only MWs mentioned (not obs) included cord blood collection and cord clamping.	*Limitations*: Participants were voluntary so self‐selection bias is possible and generalizability is limited. Participants predominantly from 1 region of the USA.

*Note*: *Health professional*: Obs—obstetricians; MWs—midwives; FP—family physicians. *Clamping practice*: UCC—umbilical cord clamping; DCC—deferred cord clamping; ECC—early cord clamping. *Other*: PPH—postpartum hemorrhage.

Maternity health professionals' knowledge, attitudes, and/or practice of umbilical cord clamping were explored internationally using studies from: the UK,^32^, ^33^, ^35^, ^36^ North America,^11^, ^15^, ^39^ Canada,^14^, ^38^, ^40^ Albania,^34^ Peru,^37^ Spain,^43^ Saudi Arabia,^44^ Tanzania,^42^ and Australia.^41^, ^45^ One international study involving obstetricians from Europe, the UK, North America, Canada, and Australia was also included.^16^

We summarize the eligible papers included in this integrative review, including aim, setting, sample, design, findings, limitations, and strengths, in Table 2.

## 3 RESULTS

Four main subject areas pertaining to maternity health professionals' knowledge, attitudes, or practices of umbilical cord clamping were identified: a) knowledge of optimal cord clamp timing; b) attitudes and perceptions of early vs deferred cord clamping; c) cord clamping practice; and d) rationale for cord clamping practice. These were grouped into disciplinary domains for midwives, obstetricians, or both professional groups where the findings from studies did not distinguish. Studies that reported on family physicians have been incorporated with obstetricians.^11^, ^14^, ^38^, ^39^, ^40^, ^45^

All aspects of third‐stage labor management were reported and discussed in six papers included in this review. One paper^43^ also reported and discussed cord blood milking, cord blood banking, and cord blood donation. In accordance with the primary aim of this review, only cord clamp timing was analyzed.

### 3.1 Knowledge and awareness of optimal cord clamping time

Midwives' knowledge and awareness of optimal cord clamping time were reported in five papers.^33^, ^35^, ^36^, ^40^, ^45^ The most common questions focused on familiarity and/or knowledge of current guidelines, definitions of ECC and DCC, and associated risks and benefits of both. Midwives were found to be very knowledgeable about current clinical guidelines for third‐stage labor management including CCT,^40^, ^45^ but variations were found in their definition of what constituted ECC and DCC. The majority of midwives defined ECC for term infants as immediately or within one minute of birth,^33^, ^35^, ^45^ and DCC as once cord pulsations ceased.^33^, ^35^, ^36^, ^45^

Obstetricians' knowledge and awareness of optimal cord clamping time was reported in six studies.^15^, ^16^, ^35^, ^36^, ^40^, ^45^ Obstetricians, such as midwives, were familiar with current guidelines that at the time included ECC as part of third‐stage labor management.^40^ Over half of study participants self‐reported they were unaware of optimal CCT,^15^ and a small number reported being unaware of evidence that supported DCC.^16^ Definitions of ECC timing were similar to those of midwives, with the majority of obstetricians defining ECC as under one minute,^35^, ^36^, ^45^ and DCC as after cord pulsations ceased.^35^, ^36^

Findings of midwives and obstetricians' knowledge about benefits and risks associated with ECC and DCC were combined in two papers.^14^, ^36^ High iron stores and reduced risk of anemia were the most frequently reported benefits of DCC in both term and preterm infants.^14^ Jaundice, polycythemia, and delay in resuscitation were the most commonly cited risks.^14^, ^36^ Reasons for ECC in term infants included resuscitation or obstetric intervention, and prevention of maternal blood loss as a reason for ECC in preterm infants.^14^

### 3.2 Attitudes, opinions, and perceptions of early vs deferred cord clamping

Midwives' attitudes and perceptions of CCT were reported in six papers.^11^, ^33^, ^40^, ^41^, ^42^, ^45^ Expectant women's preferences were significant factors in the management of third‐stage labor and cord clamp timing.^11^, ^33^, ^45^ In an early study of third‐stage labor management, midwives disagreed with, or rejected, ECC guidelines as part of AMTSL management.^40^ In a recent study, midwives expressed that cord clamping practice should be reframed, with DCC being regarded as the accepted practice; ECC should be considered the variation to standard practice. This stance reflects midwives' perceptions of the positive impact DCC has on the health and well‐being of the infant.^41^ However, it should be noted that whereas it is commonly perceived that DCC results in a time‐dependent net placental‐to‐infant blood transfusion, whether or not this occurs and to what extent is also likely dependent on the infant's physiological state as opposed to length of time between birth and cord clamping alone.^46^

Obstetricians' attitudes or perceptions of CCT were also reported in six papers.^15^, ^16^, ^40^, ^41^, ^42^, ^45^ In contrast to midwifery findings, almost all obstetricians and family physicians agreed with guidelines that included ECC.^16^, ^40^ These differences may be due, at least in part, to obstetricians being more focused on avoiding and responding to maternal and infant complications such as postpartum hemorrhage, resuscitation, and the need for infant cord blood gas collection.^27^

Combined findings for midwives and obstetricians' attitudes and perceptions to CCT were reported in five papers.^32^, ^35^, ^36^, ^40^, ^44^ Benefit of ECC was questioned by both groups; many did not believe there was an association between ECC and postpartum hemorrhage.^32^ In comparison, most obstetricians, family physicians, and midwives in an earlier study agreed or strongly agreed that AMTSL, *including early clamping,* was supported by research and was evidence‐based.^40^ This attitude has changed, as a more recent study found that a majority among all professional groups believe that DCC is beneficial for both term and preterm infants, including improved long‐term neurological development.^44^

### 3.3 Cord clamping practices

Midwives' cord clamp practices were reported in 13 papers,^14^, ^33^, ^35^, ^36^, ^37^, ^38^, ^39^, ^40^, ^41^, ^42^, ^43^, ^44^, ^45^ and DCC was the preferred practice.^14^, ^35^, ^36^, ^37^, ^39^, ^40^, ^45^ Most midwives made a conscious decision about CCT in term infants, self‐reporting DCC by at least 2 minutes, with fewer tending toward DCC in a preterm infant.^14^, ^39^, ^40^, ^45^ Self‐reported cord clamp practices varied from that observed in practice with most midwives clamping earlier than stated.^33^, ^35^ The median time for clamping was around 81 seconds.^38^ Midwifery education and training on cord clamping best practice was demonstrated to greatly increase the practice of DCC among midwives.^37^

Obstetricians' cord clamp practices were also reported in 13 papers.^14^, ^16^, ^34^, ^35^, ^36^, ^38^, ^39^, ^40^, ^41^, ^42^, ^43^, ^45^ ECC was predominant practice. Most obstetricians and family physicians self‐reported to clamp the cord in under 30 seconds.^14^, ^16^, ^35^, ^38^, ^40^ Some obstetricians self‐reported cord clamping times to be longer at around one minute,^36^, ^39^ but when their practice was observed, CCT were less than they reported.^34^ However, in a recent study, the majority of the combined professional cohort self‐reported DCC after pulsations ceased.^43^

Sivaraman and Arulkumaran (2011) conducted a survey in the UK and identified that despite obstetricians and midwives knowing some of the disadvantages of ECC, most self‐reported to clamp within 40 seconds. Although midwives comprised two thirds of the sample (n = 100/148), in this study findings still favored ECC. This contrasts with results reported previously in studies involving midwife‐only samples in other British settings, and internationally.^14^, ^35^, ^36^, ^37^, ^39^, ^40^

### 3.4 Reasons for cord clamping practice

The rationale for health professional cord clamping practices was reported in 12 studies.^11^, ^14^, ^15^, ^16^, ^33^, ^36^, ^38^, ^40^, ^41^, ^42^, ^44^, ^45^ The most common factors midwives cited for cord clamping practices were women's preference, infant resuscitation, training, guidelines, and experience.^11^, ^14^, ^36^, ^40^, ^41^, ^45^ In addition, the presence of a second midwife influenced practice, as did speed of birth.^33^ Median clamp times were more likely to be longer with uncomplicated spontaneous, low‐risk births, when cord blood was not collected.^38^

Obstetricians cited infant and maternal complications such as resuscitation, placental abruption, and placenta previa as the main reasons for not adhering to evidence‐based DCC recommendations,^14^, ^15^, ^36^ along with difficulties achieving DCC in practice.^16^ Professional training, evidence‐based medicine, clinical guidelines, and own experience were also cited for why ECC might be engaged as part of AMTSL.^40^ Those who did not clamp early were more likely to cite own experience, professional training, and risk assessment as their rationale.^40^ A more recent study revealed 26% of the surveyed maternity health professional participants cited no specific reason for their cord clamping practice.^44^

## 4 DISCUSSION

This integrative review identified and reviewed studies of maternity health care professionals' knowledge, attitudes, and practices around cord clamp timing in response to changing CCT recommendations. Similarities and differences between professional groups were explored, and factors influencing practice were compared.

Uncertainty among maternity health professionals as to optimal CCT, and definitions of ECC and DCC are key findings. Guideline variation and lack of standardized definitions of ECC and DCC timing make it difficult for policy writers and health care organizations to implement evidence‐based guidelines. This in turn hinders health professionals in providing well‐informed, consistent information to parents about third‐stage labor care options, in particular CCT. To address this, the development of cord clamping care bundles could be developed. Safety bundles do not entail a set of evidence‐based recommendations for practice and care processes known to improve outcomes.^47^ Bundles are not a new guideline but rather represent a selection of existing guidelines and recommendations in a form that aids the implementation and consistency of practice.^48^

Recommendations for cord clamp timing have changed over time. ECC was originally recommended practice for AMTSL, but evidence now suggests this is not optimal practice and many guidelines internationally reflect this evidence.^49^, ^50^, ^51^ Increasingly, DCC is advocated as best practice,^52^ yet high‐quality evidence on CCT is limited, making it difficult to standardize practice, as reflected in the practices found here.

Although the findings relating to health professional understanding of cord clamping practices were included in this review, most studies did not differentiate between health professional CCT in preterm and term infants, and vaginal vs cesarean births. This may have been because of limitations of the tools used or generalized approaches used in reporting of results.

We identified attitudes and opinions about CCT differed among the two professional groups. Midwives tended to be guided by patient preference and choice, whereas obstetricians' opinions on CCT were guided by the need for resuscitation and obstetric intervention.^11^, ^16^, ^33^, ^45^ Midwives initially tended to disagree or reject ECC as part of AMTSL, whereas obstetricians supported and agreed with this component.^40^ These differences may be related to experience, and arguably that experienced staff may be more opinion‐based in their care rather than evidence‐based.^53^

Similarities exist between the groups about rationale for cord clamp practice. All maternity professionals reported that training, guidelines, experience, the need to resuscitate, or obstetric intervention influenced practice. The major difference between the two groups was that midwives were predominantly guided by women's preference for CCT,^11^, ^14^, ^36^, ^40^, ^45^ a factor not reported by obstetricians. This may be attributed to midwifery philosophy of women‐centered care, which promotes respecting the woman's ownership of her health information, rights, and preferences while protecting her dignity and empowering her choices.^54^ The different scope of practice of the professional groups may also contribute to this variation, as midwives tend to care for low‐risk women, whereas obstetricians are more likely to care for higher risk women where intervention is required.

Both groups of maternity health professionals agreed that further research was needed on optimal cord clamp timing, with participants stating current guidelines were inadequate and additional evidence was required to guide practice.^35^, ^36^, ^41^ Evidence to date has revealed DCC benefits in term and preterm infants.^6^, ^9^, ^50^ Further clarity around benefits of DCC and use of appropriate education strategies that link research with the promotion of quality care may assist with the development, implementation, and adherence of clear guidelines for health professional clinical practice and client education.^55^

Although we identified in this integrative review that midwives clamp later than obstetricians, self‐reported clamping times seemed to differ from what was identified in observational studies. Midwives self‐reported DCC as their most common practice,^14^, ^39^, ^40^, ^45^ and obstetricians reported their most common practice as ECC.^14^, ^16^, ^35^, ^38^, ^40^ However, when practice was observed in studies, both groups tended to clamp earlier than they stated.^33^, ^34^ This could be attributed to the Hawthorn Effect whereby clinicians become more cognizant of their behaviors and may subsequently change them while under observations.^56^ As such, additional studies may not be a true reflection of cord clamping practice. Regardless, this reinforces the need for clearer guidance and consistency as to optimal CCT.

Knowledge deficits of current available evidence about third‐stage labor and neonatal transition were identified.^36^ Obstetricians who acknowledged that DCC was important had opinions about risks and benefits that were inconsistent with current literature.^15^ Even when health professionals were aware of DCC benefits, and it was indicated, they continued to practice ECC.^32^ This finding highlights the conservative nature of practice and the substantial influence that traditional practices have on the ability of practitioners to adapt and change in response to new evidence‐based practices.^36^

### 4.1 Strengths and limitations

The use of an integrative review approach facilitated inclusion of a diverse range of qualitative and quantitative studies relating to maternity health professionals' knowledge, attitudes, and practice of CCT. Although the scope of this review focused on term infants, we recognize that health professional practices of cord clamping maybe be influenced in cases of preterm and mode of birth^57^ and these factors affecting CCT warrant further review. This integrative review was also limited to studies published in English.

The papers included in this review varied significantly in sample size (n = 22 to 1243), with some very small studies included; however, small sample sizes were appropriate to the qualitative design used.^11^, ^41^, ^42^ Some survey tools to measure knowledge, attitudes, and practices were not consistent between studies, were poorly described, or were not validated.^15^, ^16^, ^32^, ^36^, ^43^ Self‐reporting of practices in studies may also be an unreliable^14^, ^32^, ^35^, ^36^, ^39^, ^40^, ^43^, ^44^ representation of clinical practice.

The findings of some papers were context‐specific and may not be generalized to other settings,^33^, ^34^, ^36^, ^37^, ^38^, ^44^, ^45^ or reported on third‐stage management inclusive of cord clamping, but not specifically CCT.^11^, ^34^, ^35^, ^36^, ^39^, ^40^, ^41^ Publication date limits were set over a 13‐year period, during which time study findings and cord clamp recommendations changed considerably, highlighting the need to further investigate health professional awareness and uptake of current evidence.

### 4.2 Conclusions

Cord clamp timing is a decision made at every birth, and the consequences of this decision have both short‐term and long‐term implications for infant health. CCT is an important part of third‐stage labor care, and midwives and obstetricians hold different opinions and practices around CCT. In this integrative review, we also identified significant gaps in health professionals' knowledge pertaining to optimal CCT, including current evidence in support of DCC. There is a need for professional and educational bodies to ensure current evidence informs guidelines for clinical practice, and research on the benefits of DCC must be embedded into midwifery and obstetric education curricula and professional development programs.^58^ Cord clamp timing requires intentional discourse between health professionals and expectant parents, to enable parents to be actively involved in care and informed decision making about their preferred options for CCT, and management of cord blood at birth.

## Supporting information


File S1
