# Supplementary material for: Integrated review of the knowledge, attitudes, and practices of maternity health care professionals concerning umbilical cord clamping
Source: Birth. 2022 May 18;49(4):595–615. doi: 10.1111/birt.12647 (PMC9790596; doi:10.1111/birt.12647)
Supplement: Supplementary file 1 — File S1 [file BIRT-49-595-s001.docx]

**Supplemental Table A: Appraisal of Quantitative studies by study design using CASP tools (CASP, 2018)**

| Quantitative Studies | | | | | | | | | | | | | | |
| --- | --- | --- | --- | --- | --- | --- | --- | --- | --- | --- | --- | --- | --- | --- |
|  | Are the results of the study valid? | | | | | | | | What are the results? | | Will the results help locally? | | |  |
| Article no. (Ref) | Did the study address a clearly focused issue? | Did the authors use appropriate methods to answer their question? | Was the cohort recruited in an acceptable way? | Was exposure measured to minimise bias? | Was outcome measured to minimise bias? | Have the authors identified all confounding factors? | Have they taken account of the confounding factors in the design and/or analysis? | Was follow up complete? | Are results presented transparently and precisely? | Are the results plausible? | Can the results be applied to the local population? | Do the results fit with other evidence? | Does this study have direct implications for practice? | CASP items addressed |
| Ortiz-Esquinas et al (2020) | Yes | Yes | Yes | Yes | Yes | Yes | Yes | Yes | No | No | Yes | Yes | Yes | 12/13 |
| Ibrahim et al (2017) | Yes | Yes | Yes | Yes | Yes | Yes | Yes | Yes | No | No | Yes | Yes | Yes | 12/13 |
| 1 Schorn et al (2017) | Yes | Yes | Yes | Yes | Yes | Yes | Yes | Yes | Yes | Yes | Yes | Yes | Yes | 13/13 |
| 3 Jelin et al (2014) | Yes | Yes | Yes | Yes | Yes | Yes | Yes | Yes | Yes | Yes | Yes | Yes | Yes | 13/13 |
| 4 Stoll & Hutton (2012) | Yes | Yes | Yes | Yes | Yes | Yes | Yes | Yes | Yes | Yes | Yes | Yes | Yes | 13/13 |
| Hutton et al (2012) | Yes | Yes | Yes | Yes | Yes | Yes | Yes | Yes | Yes | Yes | Yes | Yes | Yes | 13/13 |
| 13 Blouin et al (2011) | Yes | Yes | Yes | Yes | Yes | Yes | Yes | Yes | Yes | Yes | Yes | Yes | Yes | 13/13 |
| 5 Downey & Bewley (2010) | Yes | Yes | Yes | Yes | Yes | Yes | Yes | Yes | Yes | Yes | Yes | Yes | Yes | 13/13 |
| 6 Farrar et al (2010) | Yes | Yes | Yes | Yes | Yes | Yes | Yes | Yes | Yes | Yes | Yes | Yes | Yes | 13/13 |
| 7 Bimbashi et al (2010) | Yes | Yes | Yes | Yes | Yes | Yes | Yes | Yes | Yes | Yes | Yes | Yes | Yes | 13/13 |
| 8 Sivaraman & Arulkumaran (2011) | Yes | Yes | Unsure | Unsure | Unsure | Unsure | Unsure | Yes | Unsure | Yes | Yes | Yes | Yes | 7/13 |
| 2 Ononeze & Hutchon (2009) | Yes | Yes | Yes | Yes | Yes | Yes | Yes | Yes | No | Yes | Unsure | Yes | Yes | 11/13 |
| 9 Tan et al (2008) | Yes | Yes | Yes | Yes | Yes | Yes | Yes | Yes | Yes | Yes | Yes | Yes | Yes | 13/13 |
| 10 Airey et al (2008) | Yes | Yes | Yes | Yes | Yes | Yes | Yes | Yes | Yes | Yes | Yes | Yes | Yes | 13/13 |

**Supplemental Table B: Appraisal of Qualitative studies by study design using CASP tools (CASP, 2013)**

| Qualitative Studies | | | | | | | | | | | |
| --- | --- | --- | --- | --- | --- | --- | --- | --- | --- | --- | --- |
| Article no. (ref) | Was there a clear statement of the aims of the research? | Is qualitative methodology appropriate? | Was design appropriate to address the aims? | Was the recruitment strategy appropriate to match aims? | Were the data collected in a way that addresses the research issue? | Has the relationship between researcher and participants been adequately considered? | Have ethical issues been considered? | Was the data analysis sufficiently rigorous? | Is there a clear statement of findings? | Is the research valuable? | Validity score |
| Peberdy et al (2020) | Yes | Yes | Yes | Yes | Yes | Yes | Yes | Yes | Yes | Yes | 10/10 |
| Mwakawanga & Mselle (2020) | Yes | Yes | Yes | Yes | Yes | Yes | Yes | Yes | Yes | Yes | 10/10 |
| 11 Schorn et al (2015) | Yes | Yes | Yes | Yes | Yes | Yes | Yes | Yes | Yes | Yes | 10/10 |

CASP. 2018. *Critical Appraisal Skills Programme (CASP): Making sense of evidence.* [Online]. https://casp-uk.net/casp-tools-checklists/ [Accessed November 11 2020].
